# Regional Homogeneity and Multivariate Pattern Analysis of Cervical Spondylosis Neck Pain and the Modulation Effect of Treatment

**DOI:** 10.3389/fnins.2018.00900

**Published:** 2018-12-06

**Authors:** Jun Chen, Zengjian Wang, Yiheng Tu, Xian Liu, Kristen Jorgenson, Guoxi Ye, Chenlin Lin, Jianhua Liu, Joel Park, Courtney Lang, Bo Liu, Jian Kong

**Affiliations:** ^1^Department of Radiology, Guangdong Provincial Hospital of Chinese Medicine, Guangzhou, China; ^2^Department of Maternal and Child Health, School of Public Health, Sun Yat-sen University, Guangzhou, China; ^3^Department of Psychiatry, Massachusetts General Hospital, Harvard Medical School, Charlestown, MA, United States; ^4^Key Laboratory for Studying Regularities and Mechanism of Acu-moxibustion, Department of Acu-moxibustion, Guangdong Provincial Hospital of Chinese Medicine, Guangzhou, China

**Keywords:** chronic neck pain, temporo-parietal junction, cervical spondylosis, regional homogeneity, resting-state functional connectivity

## Abstract

**Objects:** We investigated brain functional alteration in patients with chronic cervical spondylosis neck pain (CSNP) compared to healthy controls (HCs) and the effect of intervention.

**Methods:** 104 CSNP patients and 96 matched HCs were recruited. Patients received 4 weeks of treatment. Resting-state fMRI and Northwick Park Neck Pain Questionnaire (NPQ) were collected before and after treatment. Resting state regional homogeneity (rs-ReHo) and multivariate pattern analysis (MVPA) were applied to (1) investigate rs-ReHo differences between CSNP patients and controls and the effect of longitudinal treatment and (2) classify CSNP patients from HCs and predict clinical outcomes before treatment using MVPA.

**Results:** We found that (1) CSNP patients showed decreased rs-ReHo in the left sensorimotor cortex and right temporo-parietal junction (rTPJ), and rs-ReHo at the rTPJ significantly increased after treatment; (2) rs-ReHo at rTPJ was associated with NPQ at baseline, and pre- and post-treatment rs-ReHo changes at rTPJ were associated with NPQ changes in CSNP patients; and (3) MVPA could discriminate CSNP patients from HCs with 72% accuracy and predict clinical outcomes with a mean absolute error of 19.6%.

**Conclusion:** CSNP patients are associated with dysfunction of the rTPJ and sensorimotor area.

**Significance:** rTPJ plays on important role in the pathophysiology and development of CSNP.

**HIGHLIGHTS**

- CSNP patients showed decreased ReHo in the left S1/M1 and right TPJ.
- ReHo at rTPJ increased after treatment in CSNP patients.
- ReHo at rTPJ and S1/M1 can classify CSNP patients and HCs.
- ReHo at baseline can predict NPQ changes after treatment.

## Introduction

Cervical spondylosis (CS) is a common age-related chronic disease characterized by stiffness and neck and upper back pain. Chronic CS neck pain (CSNP) has become an important public health and social issue because of its high prevalence, unsatisfactory treatment options, large medical burden, and reduction in quality of life (Binder, 2007; Hoy et al., 2010; Baime, 2016). Recent studies have demonstrated that patients with cervical spondylotic myelopathy (CSM), the later stage of CS, have cerebral cortex reorganization as a result of spinal cord compression (Holly et al., 2007; Dong et al., 2008; Holly, 2009; Duggal et al., 2010). However, the characteristics of cortical activity in CSNP patients without spinal cord compression have not been reported.

Previous studies showed that patients with spinal cord compression in CSM are associated with increased activation in the primary motor and premotor cortices and activation loss in the sensory cortex (Kowalczyk et al., 2012; Zhou et al., 2014; Zhou F. et al., 2015; Zhou F. Q. et al., 2015). Single-photon emission computed tomography (SPECT) and positron emission tomography (PET) studies found significant abnormal hypoperfusion and hypometabolism in brain regions such as the bilateral parieto-occipital cortex in chronic neck pain patients with whiplash syndrome (Otte et al., 1995, 1996, 1997a,b; Bicik et al., 1998; Lorberboym et al., 2002; Guez, 2006; Linnman et al., 2009). Neuroimaging studies also found changes in brain structure and function in chronic back pain (CBP) patients (Wand et al., 2011; Wasan et al., 2011; Kong et al., 2013; Loggia et al., 2013; Mao et al., 2014; Yu et al., 2014a; Pijnenburg et al., 2015; Hotz-Boendermaker et al., 2016).

Recent studies also found improvements in brain functional activity and connectivity in patients with spinal cord decompression following intervention (Holly et al., 2007; Dong et al., 2008; Holly, 2009; Duggal et al., 2010; Tam et al., 2010; Hrabalek et al., 2014; Green et al., 2015; Hrabalek et al., 2015; Tan et al., 2015; Bhagavatula et al., 2016). Dong and colleagues found that abnormalities in the cortical sensorimotor recruitment pattern in CSM patients gradually disappeared and were accompanied by behavioral improvements after surgery (Dong et al., 2008). Effective longitudinal treatment can also modulate the aberrant functional neural activity and brain structure of chronic low back pain patients (Lorimer Moseley, 2005; Baliki et al., 2008; Seminowicz et al., 2011; Younger et al., 2011; Li et al., 2014; Ceko et al., 2015; Louw et al., 2015; Shi et al., 2015; Vrana et al., 2015; Braden et al., 2016). Nonetheless, there is a lack of research evaluating the modulation effect of effective treatment on the brain activity of CSNP patients.

In recent years, machine learning has gained popularity in the translational neuroimaging community for it allows researchers to identify brain signatures of patients with psychiatric and neurologic diseases and make accurate classifications (Arbabshirani et al., 2017). A widely applied machine learning approach, multivariate pattern analysis (MVPA) uses pattern classifiers to capture the information encoded by spatially correlated voxels in fMRI and has proven to be more sensitive to the functional organization of the cortex (e.g., general linear model; Haxby, 2012). This technique has been used to explore the neurophysiology of chronic pain disorders, including migraine (Chong et al., 2017), chronic low back pain (Ung et al., 2014) and fibromyalgia (Lopez-Sola et al., 2017).

In this study, we explored (1) the differences in resting state brain activity between CSNP patients and matched healthy controls (HCs), (2) how longitudinal treatment can modulate brain abnormalities in CSNP patients using regional homogeneity (ReHo), and (3) if MVPA approaches can classify CSNP patients and HCs and predict clinical outcomes before treatment. We hypothesized that (1) CSNP patients would be associated with altered activity in specific brain regions (e.g., sensorimotor cortex or other regions) compared with HCs, (2) symptom reduction after an effective treatment could modulate the altered brain activities of CSNP patients, and (3) CSNP patients and HCs could be discriminated using MVPA.

## Materials and Methods

### Participants

This study was approved by the Ethics Committee at the Second Affiliated Hospital of Guangzhou University of Chinese Medicine. The experiment was performed in accordance with approved guidelines. Participants were recruited from the outpatient clinics of the Second Affiliated Hospital, Guangzhou University of Traditional Chinese Medicine. This study was registered on http://www.chictr.org.cn/index.aspx (ChiCTR-IPR-14005282). Written informed consent was obtained from each participant. Subjects were recruited from July 2010 to December 2012.

The inclusion criteria were as follows: (1) a confirmed diagnosis of CSNP in accordance with the diagnostic criteria published by the Chinese Association of Rehabilitation (2010) and in accordance with the International Classification of Diseases, 10th edition (ICD-10) codes^1^; (2) male or female 18–35 years of age; (3) no signs of musculoskeletal system abnormalities upon physical examination; (4) not receiving any treatment within the last 7 days; and (5) informed consent obtained. Patients were excluded if they met any of the following criteria: (1) history of neck trauma; (2) vertebral body or spinal canal cancer, tuberculosis, or severe osteoporosis; (3) history of neck surgery or presence of congenital malformation of the cervical vertebrae; (4) pregnant or breastfeeding (females); and (5) presence of a severe systemic disease such as tumors, diabetes mellitus, kidney disease, or digestive system disease.

Additionally, pain-free age and gender-matched individuals were recruited for this study as HCs. Each subject underwent a medical history evaluation and physical examination.

### Interventions

All CSNP patients received acupuncture treatments for one month at the Second Affiliated Hospital of Guangzhou University of Chinese Medicine. The CSNP patients were assigned to one of five acupuncture treatment groups based on the acupuncture points applied. The acupoints selected were bilateral Bailao (EX-HN15) in acupuncture group 1 (AG1), bilateral EX-HN15 and Hegu (LI4) in AG2, bilateral EX-HN15 and Zhongzhu (TE3) in AG3, bilateral LI4 in AG4, and bilateral Zusanli (ST36) in AG5.

Each patient received about 30 min of acupuncture treatment approximately once every three days for a total of 10 sessions over a 4-week period. The needles were inserted to a depth of 20–30 mm, perpendicular to the surface of the skin. Electroacupuncture was performed to stimulate the relevant acupoints for 30 min using a continuous-wave, 1-Hz frequency, and a comfortable strength until *deqi* sensation (soreness, numbness, distention, and heaviness) was obtained.

The Northwick Park Neck Pain Questionnaire (NPQ) was administered to CSNP patients to assess neck pain and disability (Leak et al., 1994). The NPQ scores were assessed before the first treatment as baseline data and again after the final treatment by the patients themselves following thorough instruction from the researchers.

### Image Acquisition

All participants underwent a structural and functional MRI scan using a 3.0T MR system (Magnetom Verio, Siemens, Germany) at the Department of Radiology at the Second Affiliated Hospital of Guangzhou University of Chinese Medicine. Cushions were used to support participants’ heads within the coil to prevent excessive head motion. Participants were asked to stay still with their eyes closed. All images were acquired parallel to the anterior-commissure-posterior-commissure line with an auto-align technique. The functional data were collected using an echo-planar imaging sequence: 31 axial slices; repetition time (TR) = 2000 ms; echo time (TE) = 30 ms; slice thickness = 3.5 mm; gap = 0.35 mm; flip angle (FA) = 90°; matrix = 64 × 64; field of view (FOV) = 224 mm × 224 mm, 240 time points. 3D structural images were acquired using a T1-weighted MP-RAGE sequence: 176 sagittal slices; TR = 1900 ms; TE = 2.27 ms; inversion time = 900 ms; slice thickness = 1.0 mm; no gap; FA = 9°; matrix = 256 × 256; FOV = 256 mm × 256 mm.

### Data Preprocessing

Data preprocessing was carried out using Data Processing Assistant for Resting-State fMRI (DPARSF^2^), which is based on Statistical Parametric Mapping (SPM^3^) and the toolbox for Data Processing and Analysis of Brain Imaging (DPABI^4^). The preprocessing procedures included: time alignment across slices, motion correction, within-subject registration between T1 and EPI images, T1 segmentation, and normalization of the BOLD fMRI datasets to register them to Montreal Neurologic Institute (MNI) space with voxels resampled at 3 mm × 3 mm × 3 mm. Participants were excluded if head motion exceeded 1.5 mm and 1.5°. Finally, the waveform of each voxel was passed through a band-pass filter (0.01–0.08 Hz) to reduce the effects of low-frequency drift and high-frequency physiological noise. Finally, several nuisance signals were regressed out from each voxel’s time course, including 24-parameter head-motion profiles, mean white matter (WM) and cerebrospinal fluid (CSF) time series.

### Regional Homogeneity (ReHo)

ReHo was calculated by the Kendall’s coefficient of concordance (KCC) (Kendall, 1990) using the REST toolkit^5^. Individual resting state ReHo (rs-ReHo) maps were generated by assigning each voxel a value corresponding to the KCC of its time series with its 26 nearest neighboring voxels (Zang et al., 2004). The individual rs-ReHo maps were standardized by their own mean KCC. Then, a Gaussian kernel with a full-width at half-maximum of 4 mm was used to smooth the images in order to reduce noise and residual differences.

Group analysis was calculated with a random effects model using the Data Processing and Analysis of Brain Imaging toolbox (DPABI^6^). We first compared the rs-ReHo differences between CSNP patients and HCs using two-sample *t*-tests. Then, a partial correlation analysis was used to investigate the relationship between the rs-ReHo of these aberrant brain regions and NPQ scores in the CSNP patients, controlling for age and gender as covariates using SPSS.

In order to explore whether clinical improvement after an effective treatment could affect the brain’s intrinsic neural activity in those aforementioned aberrant brain regions, we defined all brain regions that showed significant rs-ReHo changes between CSNP patients and HCs as regions of interest (ROI) and compared the pre- and post-treatment rs-ReHo changes in CSNP patients using a paired *t*-test within the ROIs as well as the whole brain. Then, partial correlation analysis was used to investigate the relationship between pre- and post-treatment rs-ReHo changes within the ROIs and corresponding NPQ score changes in the CSNP patients, controlling for age and gender as covariates using SPSS. A threshold of voxel-wise *p* < 0.005 and *p* < 0.05 family-wise error corrected at cluster level was applied for whole brain analyses, and a small volume correction method (voxel-wise *p* < 0.005 and *p* < 0.05 FWE corrected) was used for all ROIs (derived from a comparison between the CSNP patients and healthy controls before and after acupuncture treatments).

### Multivariate Pattern Analysis (MVPA)

The ROIs defined in the ReHo analyses were used for the MVPA analyses with two objectives: (1) classify CSNP patients and HCs and (2) predict treatment responses using baseline (pre-treatment) ReHo values. To avoid the risk of overfitting, all analyses were based on fivefold cross-validation (CV) (Pereira et al., 2009).

In the first step, machine learning models were trained to classify CSNP patients and HCs using ReHo values within the ROIs. A support vector machine (SVM) classifier was used and the implementation of SVM was based on LIBSVM (Chang and Lin, 2011). To quantify the performance of the SVM classifier, classification accuracy, sensitivity and specificity were calculated. Here, sensitivity and specificity represent the proportion of patients correctly classified and the proportion of HCs correctly classified, respectively. To further assess the performance of the classifier and evaluate the significance of classification accuracy, we ran permutation testing. In each test, we randomly permuted the class labels of the data prior to training. Fivefold CV was then performed on the permuted dataset and the procedure was repeated 10,000 times. If the classifier trained on real class labels had an accuracy exceeding the 95% confidence interval generated from the accuracies of the classifiers trained on randomly relabeled class labels, this classifier was considered to be well-performing.

In the second step, we aimed to predict treatment responses (percentage change of NPQ scores) using baseline ReHo values. Here, we used a novel fMRI prediction approach, namely sliced inverse regression (SIR), to extract the most predictive information from ROIs and subsequently predict responses. SIR is powerful because it can work efficiently regardless of the linearity of the relationship between fMRI data and their labels. The details of this implementation of SIR-based fMRI prediction can be found in Tu et al. (2017). Briefly, in the first step, we performed SIR on the features Φ ∈ *R^n×p^* and corresponding behavioral data ψ ∈ *R^n^* (i.e., percentage changes of NPQ), resulting in the effective dimension reduction (e.d.r.) direction β ∈ *R^p×K^* and SIR-derived feature set S = Φβ ∈ *R^n×K^*. In the second step, we used a support vector regression (SVR) ψ = *C*(*S_t_*α;*t* = 1, … ,*n*) with α ∈ *R^K^* to estimate the predicted value and the contribution of each variable in the SIR reduced space to predict ψ. In the third step, we transformed α back to the original space by multiplying β, resulting in the weights for all features 𝜃 = βα. To quantify the performance of prediction, we used the prediction-outcome correlation which is defined as the correlation between the actual and predicted differences of NPQ scores, as well as mean absolute error (MAE) (Tu et al., 2016), which is defined as

(1) $$MAE=\frac{1}{N}\sum_{n=1}^{N}|\psi_{n}-\;{\hat{\psi }}_{n}|.$$

where ψ_n_ and ${\hat{\psi }}_{n}$ denote, respectively, the actual and predicted differences of NPQ scores (1-Post NPQ/Pre NPQ) for patient *n*, and *N* is the number of CSNP patients. The number of slices for SIR was set to 5 and the number of directions was set to 4. Performance measures were assessed using permutation testing (as mentioned in the classification session).

## Results

A total of 104 right-handed CSNP patients (45 males and 59 females, age 24.90 ± 1.98 years, neck pain duration 36.49 ± 24.93 months) and 96 HCs (50 males and 46 females, age 24.80 ± 1.52 years) were recruited. There were no significant differences in age and gender between CSNP patients and HCs (*P* > 0.05).

Compared with baseline scores, all treatment groups (AG1, AG2, AG3, AG4, and AG5) showed improvement in NPQ scores (*P* < 0.01). We found no significant differences among AG1, AG2, AG3, AG4, and AG5 in NPQ score improvement (*P* > 0.05). The average NPQ score across all patients was 24.1 ± 9.2 before treatment and 12.7 ± 9.0 at the end of treatment. There was a significant decrease in NPQ scores after 1 month of treatment (*P* < 0.01).

Based on these clinical findings, we merged all acupuncture treatment groups (AAG) in the rs-ReHo analyses to investigate how an effective treatment can modulate rs-ReHo.

### ReHo Results

**FIGURE 1:**
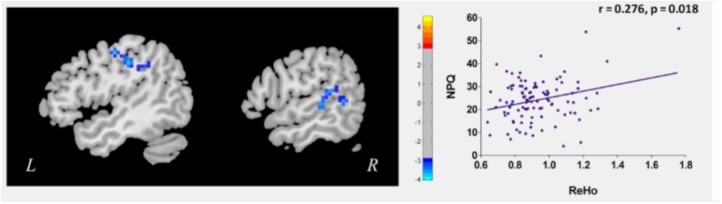
Altered resting state ReHo in CSNP patients and the modulation effect of clinical treament. The left sensorimotor cortex and rTPJ showed reduced rs-ReHo in CSNP patients compared to healthy controls. The rs-ReHo value in rTPJ was associated with neck pain intensity (NPQ) at baseline in CSNP patients.

To explore the neural pathophysiology of neck pain, we first compared all CSNP patients to HCs using a two-sample *t*-test. Compared to HCs, CSNP patients showed decreased rs-ReHo in the right temporo-parietal junction (rTPJ) and left sensorimotor cortex (left post-central gyrus and precentral gyrus) (Figure 1 and Table 1). Regression analysis indicated that the rs-ReHo in the rTPJ was positively associated with NPQ scores at baseline in CSNP patients (Figure 1).

**Table 1:** Brain regions with significant ReHo difference between CSNP patients and health controls. rTPJ, right temporo-parietal junction.

Brain region	Brodmann area	Voxels	Coordinates (MNI space)	Peak value
rTPJ/temporal cortex	22/13/21	117	60 -48 6	-3.950
left sensorimotor cortex	3/4/2	84	-51 -27 42	-4.378

We then compared post- and pre-treatment rs-ReHo differences using paired *t*-tests. Results revealed that after longitudinal clinical treatment, CSNP patients showed increased rs-ReHo in the rTPJ (peak 60, -48, 6, 13 voxels). There were no significant differences between the pre- and post-treatment rs-ReHo in other ROIs and non-ROIs at the threshold we set. Regression analysis indicated that the pre- and post-treatment rs-ReHo changes at the rTPJ were positively associated with corresponding NPQ changes in CSNP patients.

### MVPA Results

**FIGURE 2:**
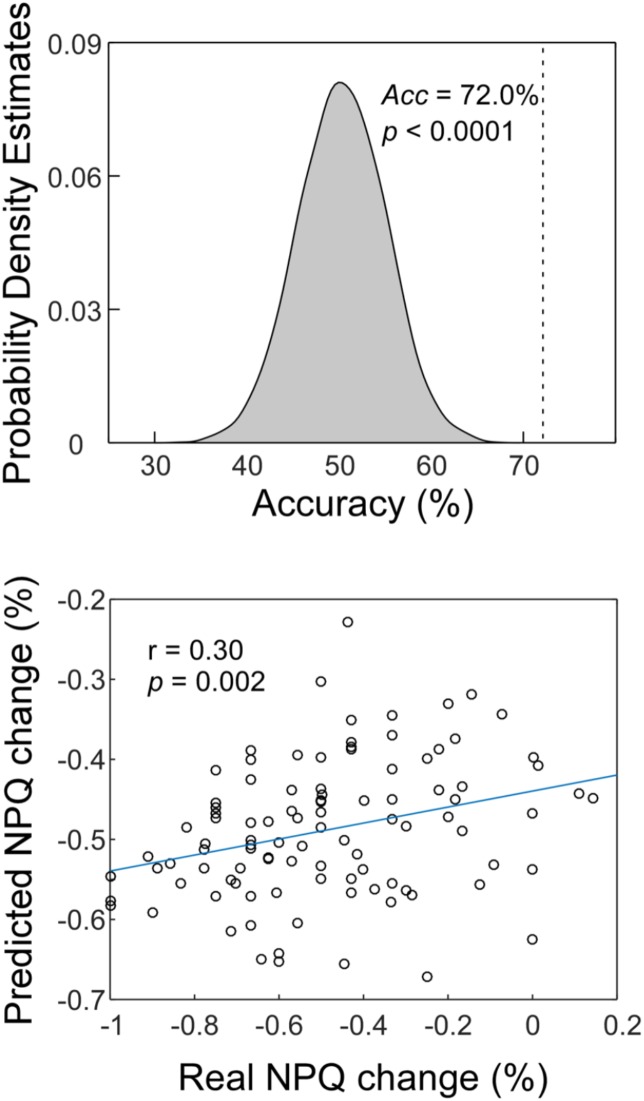
The MVPA results. *Upper panel*: The classification accuracy of discriminating CSNP patients and HCs. *Lower panel*: The prediction-outcome correlation of predicting treatment responses using baseline fMRI data.

Classification accuracy for separating CSNP patients and HCs is summarized in Figure 2, *upper panel*. Using the ReHo values within the rTPJ, left postcentral gyrus, and precentral gyrus, the classifier achieved an accuracy of 72.0% (76.0% for CSNP and 67.7% for HCs; *p* < 0.0001, permutation testing).

Similar to a previous study (Doehrmann et al., 2013), two subjects were removed as outliers from the prediction analysis since their NPQ changes were more than 3 SDs higher than the average of our samples. The correlation between actual and predicted treatment response was 0.30 (*p* = 0.002; Figure 2, *lower panel*). We obtained an MAE of 19.6% for predicting treatment responses (*p* = 0.004, permutation testing).

## Discussion

In this study, we investigated rs-ReHo differences between CSNP patients and HCs as well as how symptom relief after treatment can modulate rs-ReHo. We found that CSNP patients were associated with reduced rs-ReHo in brain regions including the rTPJ and left sensorimotor cortex as compared with HCs. In addition, we found that effective longitudinal treatment (acupuncture treatment) can normalize (increase) decreased rs-ReHo in CSNP patients. Moreover, rs-ReHo in the rTPJ was associated with neck pain intensity, as measured by NPQ scores at baseline. Following treatment, rs-ReHo changes at the rTPJ were associated with alleviation of neck pain intensity (i.e., decrease in NPQ scores) in CSNP patients. We applied MVPA for discriminating CSNP patients from HCs with an accuracy of 72% as well as predicting treatment responses using baseline data with an error of 19.6%.

ReHo has been successfully used to detect local abnormalities in subjects with different disorders such as chronic pain (Wang et al., 2014, 2015; Ke et al., 2015; Huang et al., 2016; Zhang et al., 2016) and psychiatric disorders (Liang et al., 2013; Gao et al., 2014; Wang et al., 2015; Yang et al., 2015, 2016). Compared to other resting state functional connectivity (rs-FC) methods such as seed-based rs-FC analysis or independent component analysis, ReHo can target key regions involved in the pathophysiology of neck pain rather than elucidating the connectivity between and among different regions (Yu et al., 2014b).

Neck pain accompanied by neck stiffness and discomfort is the earliest symptom of CS. Patients who do not receive appropriate treatment in the initial stage of CS later develop a complex series of clinical symptoms including spinal cord compression (i.e., CSM). Recently, studies have investigated brain functional changes in CSM (Kowalczyk et al., 2012; Zhou et al., 2014; Zhou F. et al., 2015; Zhou F. Q. et al., 2015) and found patients with CSM are associated with cortical reorganization in the primary motor cortex (Holly et al., 2007; Duggal et al., 2010; Tam et al., 2010; Kowalczyk et al., 2012; Bhagavatula et al., 2016), primary somatosensory cortex (Duggal et al., 2010; Bhagavatula et al., 2016), and sensorimotor cortex (Dong et al., 2008; Zhou et al., 2014; Tan et al., 2015; Zhou F. Q. et al., 2015).

Consistent with previous findings, we found CSNP patients were associated with reduced rs-ReHo in the sensorimotor cortex compared to HCs. This result is also consistent with previous studies that report alterations in sensorimotor cortices in conjunction with clinical deterioration in CSM patients (Dong et al., 2008; Tan et al., 2015). Some studies have found that sensorimotor cortices normalized with the recovery of function following surgical spinal cord decompression in CSM patients (Holly et al., 2007; Dong et al., 2008; Duggal et al., 2010; Tam et al., 2010; Green et al., 2015; Tan et al., 2015; Bhagavatula et al., 2016). In our study, we did not find any significant rs-ReHo changes in the sensorimotor cortex after treatment. We speculate these differences might be due to the different stages of CS investigated in the two studies. Patients with CSM often show motor and sensory deficits, while CSNP patients often show discomfort of the neck including chronic neck pain and stiffness.

In the present study, decreased ReHo in the sensorimotor cortex was mainly localized to S1. Previous studies have indicated that S1 plays a crucial role in nociceptive processing pathways in the human brain (Bushnell et al., 1999; Ploner et al., 1999). In our previous study we also found that chronic low back pain patients have increased cortical thickness and increased volume in S1 and decreased voxel-by-voxel functional connectivity during low pain conditions at S1 compared to HCs (Kong et al., 2013). Our results provide further evidence of rs-ReHo changes in the sensorimotor cortex in CSNP patients, and we speculate this may reflect aberrant central processing of neck pain afferentiation.

We also found reduced rs-ReHo in the rTPJ, including the posterior end of the right superior temporal gyrus and right inferior parietal lobule, in CSNP patients compared to HCs. Our findings were in line with previous studies that reported abnormal structure and function in the TPJ for various chronic pain conditions. It is worth noting that the literature sometimes uses the superior temporal gyrus and inferior parietal lobule instead of the TPJ. For instance, Friebel et al. (2011) used coordinate-based meta-analysis to find that patients with chronic persistent neuropathic pain were associated with activation of the right inferior parietal lobe, a component of the TPJ.

The TPJ is an approximate term that generally refers to the regions including the posterior end of the superior temporal gyrus, the inferior parietal lobule, and the lateral occipital gyrus in the human brain (Mars et al., 2012; Krall et al., 2015). Studies have suggested that the TPJ is an area in the mirror neuron system, which maintains a coherent representation of our own, as well as others’, body (Iacoboni and Dapretto, 2006; Tsakiris et al., 2008; Rizzolatti and Sinigaglia, 2010) and is also involved in imitative motor learning and control (Buccino and Riggio, 2006; Fogassi, 2011).

The mirror neuron system also participates in pain perception (Hojat and Cohen, 2012) and includes the brain regions associated with pain experience, such as the cingulate and insular cortices (Ramachandran and Oberman, 2006). Studies have suggested that the TPJ receives information from the thalamus and limbic system and projects to many other brain areas associated with pain perception and modulation, such as the lateral anterior prefrontal cortex, ventral prefrontal cortex, anterior insula, posterior cingulate, and anterior medial prefrontal cortex (Mars et al., 2012). In our study, we found that effective longitudinal acupuncture treatments can increase reduced rs-ReHo in the rTPJ, and this normalization is associated with a reduction in neck pain intensity in CSNP patients. More studies are needed to explore the role of the TPJ in the development of CSNP.

Machine learning techniques have been widely applied in translational neuroimaging studies to provide a basis for identifying neuropathological features of different diseases and show potential clinical utility beyond current clinical diagnostic categories (Pereira et al., 2009; Arribas et al., 2010; Orru et al., 2012; Zeng et al., 2012; Abi-Dargham and Horga, 2016; Woo et al., 2017). The earliest work identified brain signatures for neurological diseases, particularly Alzheimer’s Disease and related dementia (Kippenhan et al., 1992). The studies have been extended to other neurological and psychiatric conditions, such as Parkinson’s disease (Tang et al., 2010), major depression (Zeng et al., 2012), schizophrenia (Shen et al., 2010), and ADHD (Hart et al., 2014). Most studies have focused on identifying brain signatures for discriminating patients from HCs and consequently established a meaningful neurophysiological basis for the disorder of interest. Another machine learning model uses a patient’s brain measurements to predict treatment outcomes. Most focus on depression and anxiety disorders (Doehrmann et al., 2013; Hahn et al., 2015; Whitfield-Gabrieli et al., 2016) as well as schizophrenia (Sarpal et al., 2016) and Parkinson’s disease (Ye et al., 2016).

To the best of our knowledge, the present study is the first attempt to classify CSNP patients from healthy controls using resting-state fMRI and a state-of-the-art machine learning approach. Therefore, it provides a possible method of using objective measures to diagnose CSNP patients. More importantly, we used baseline resting-state fMRI to predict clinical symptom changes for a longitudinal treatment with an error of 19.6% and prediction-outcome correlation of 0.30. The present approach offers a useful tool for clinicians when determining the effectiveness of acupuncture treatment before executing treatment procedures.

### Limitations

It is worth noting that the aim of this study was to investigate the brain’s neural plasticity in CSNP patients, how an effective treatment can modulate altered neural plasticity, and the association between ReHo changes and clinical symptom changes. This paradigm has been used in previous studies from our group (Li et al., 2016) and from other groups (Zhou and Jin, 2008; Rodriguez-Raecke et al., 2009; Sato et al., 2009; Fang et al., 2012). Although our results may provide some novel insights into the mechanisms underlying effective treatment, the lack of sham treatment as a control has significantly limited our ability to infer an underlying longitudinal acupuncture treatment mechanism. Additional controls should be applied in future studies to elucidate the specific effect of the corresponding treatment.

## Conclusion

We found that CSNP patients are associated with increased regional coherences at the right temporo-parietal junction and left sensorimotor cortex. Additionally, the alleviation of symptoms after treatment can normalize the reduced rs-ReHo in CSNP patients, and this normalized rs-ReHo value is also associated with a reduction in neck pain intensity. We were also able to use MVPA to discriminate CSNP patients from HCs with an accuracy of 72% as well as predict treatment responses using baseline data with an error of 19.6%. Our results demonstrate the crucial role of the rTPJ in the pathophysiology and development of non-specific neck pain.

## Author Contributions

BL and JL proposed the study and were the guarantors. JC, ZW, YT, KJ, JP, CL, and JK analyzed the data and/or wrote the original and revised drafts of the manuscript. All authors contributed to the design and/or interpretation of the study.

## Conflict of Interest Statement

The authors declare that the research was conducted in the absence of any commercial or financial relationships that could be construed as a potential conflict of interest.

## Footnotes

1

2 http://rfmri.org/DPARSF

3 http://www.fil.ion.ucl.ac.uk/spm

4 http://rfmri.org/DPABI

5 http://restfmri.net

6 http://rfmri.org/DPABI

## References

[B1] Abi-Dargham A. Horga G. (2016). The search for imaging biomarkers in psychiatric disorders. *Nat. Med.* 22 1248–1255. 10.1038/nm.4190 27783066

[B2] Arbabshirani M. R. Plis S. Sui J. Calhoun V. D. (2017). Single subject prediction of brain disorders in neuroimaging: promises and pitfalls. *Neuroimage* 145 137–165. 10.1016/j.neuroimage.2016.02.079 27012503 PMC5031516

[B3] Arribas J. I. Calhoun V. D. Adali T. (2010). Automatic Bayesian classification of healthy controls, bipolar disorder, and schizophrenia using intrinsic connectivity maps from FMRI data. *IEEE Trans. Biomed. Eng.* 57 2850–2860. 10.1109/TBME.2010.2080679 20876002 PMC2982883

[B4] Baime M. J. (2016). In chronic nonspecific neck pain, adding Alexander Technique lessons or acupuncture to usual care improved pain. *Ann. Intern. Med.* 164:JC29. 10.7326/ACPJC-2016-164-6-029 26974729

[B5] Baliki M. N. Geha P. Y. Jabakhanji R. Harden N. Schnitzer T. J. Apkarian A. V. (2008). A preliminary fMRI study of analgesic treatment in chronic back pain and knee osteoarthritis. *Mol. Pain* 4:47. 10.1186/1744-8069-4-47 18950528 PMC2584040

[B6] Bhagavatula I. D. Shukla D. Sadashiva N. Saligoudar P. Prasad C. Bhat D. I. (2016). Functional cortical reorganization in cases of cervical spondylotic myelopathy and changes associated with surgery. *Neurosurg. Focus* 40:E2. 10.3171/2016.3.FOCUS1635 27246485

[B7] Bicik I. Radanov B. P. Schafer N. Dvorak J. Blum B. Weber B. (1998). PET with 18fluorodeoxyglucose and hexamethylpropylene amine oxime SPECT in late whiplash syndrome. *Neurology* 51 345–350. 10.1212/WNL.51.2.345 9710001

[B8] Binder A. (2007). The diagnosis and treatment of nonspecific neck pain and whiplash. *Eur. Medicophys.* 43 79–89. 17369782

[B9] Braden B. B. Pipe T. B. Smith R. Glaspy T. K. Deatherage B. R. Baxter L. C. (2016). Brain and behavior changes associated with an abbreviated 4-week mindfulness-based stress reduction course in back pain patients. *Brain Behav.* 6:e00443. 10.1002/brb3.443 26925304 PMC4754498

[B10] Buccino G. Riggio L. (2006). The role of the mirror neuron system in motor learning. *Kinesiology* 38 5–15.

[B11] Bushnell M. C. Duncan G. H. Hofbauer R. K. Ha B. Chen J. I. Carrier B. (1999). Pain perception: is there a role for primary somatosensory cortex? *Proc. Natl. Acad. Sci. U.S.A.* 96 7705–7709. 10.1073/pnas.96.14.7705 10393884 PMC33605

[B12] Ceko M. Shir Y. Ouellet J. A. Ware M. A. Stone L. S. Seminowicz D. A. (2015). Partial recovery of abnormal insula and dorsolateral prefrontal connectivity to cognitive networks in chronic low back pain after treatment. *Hum. Brain Mapp.* 36 2075–2092. 10.1002/hbm.22757 25648842 PMC6869701

[B13] Chang C.-C. Lin C.-J. (2011). LIBSVmml: a library for support vector machines. *ACM Trans. Intell. Syst. Technol.* 2 1–27. 10.1145/1961189.1961199

[B14] Chinese Association of Rehabilitation (2010). *Professional Committee of Cervical Spondylosis, Chinese Association of Rehabilitation Medicine: Guidelines for Diagnosis, Treatment and Rehabilitation for Cervical Spondylosis.* Beijing: Chinese Association of Rehabilitation.

[B15] Chong C. D. Gaw N. Fu Y. Li J. Wu T. Schwedt T. J. (2017). Migraine classification using magnetic resonance imaging resting-state functional connectivity data. *Cephalalgia* 37 828–844. 10.1177/0333102416652091 27306407

[B16] Doehrmann O. Ghosh S. S. Polli F. E. Reynolds G. O. Horn F. Keshavan A. (2013). Predicting treatment response in social anxiety disorder from functional magnetic resonance imaging. *JAMA Psychiatry* 70 87–97. 10.1001/2013.jamapsychiatry.5 22945462 PMC3844518

[B17] Dong Y. Holly L. T. Albistegui-Dubois R. Yan X. Marehbian J. Newton J. M. (2008). Compensatory cerebral adaptations before and evolving changes after surgical decompression in cervical spondylotic myelopathy. *J. Neurosurg. Spine* 9 538–551. 10.3171/SPI.2008.10.0831 19035745 PMC4090101

[B18] Duggal N. Rabin D. Bartha R. Barry R. L. Gati J. S. Kowalczyk I. (2010). Brain reorganization in patients with spinal cord compression evaluated using fMRI. *Neurology* 74 1048–1054. 10.1212/WNL.0b013e3181d6b0ea 20200344

[B19] Fang Z. Ning J. Xiong C. Shulin Y. (2012). Effects of electroacupuncture at head points on the function of cerebral motor areas in stroke patients: a PET study. *Evid. Based Complement. Alternat. Med.* 2012:902413. 10.1155/2012/902413 22956979 PMC3432396

[B20] Fogassi L. (2011). The mirror neuron systemml: how cognitive functions emerge from motor organization. *J. Econ. Behav. Org.* 77 66–75. 10.1016/j.brainresbull.2008.01.016 18394524

[B21] Friebel U. Eickhoff S. B. Lotze M. (2011). Coordinate-based meta-analysis of experimentally induced and chronic persistent neuropathic pain. *Neuroimage* 58 1070–1080. 10.1016/j.neuroimage.2011.07.022 21798355 PMC8018239

[B22] Gao W. Jiao Q. Lu S. Zhong Y. Qi R. Lu D. (2014). Alterations of regional homogeneity in pediatric bipolar depression: a resting-state fMRI study. *BMC Psychiatry* 14:222. 10.1186/s12888-014-0222-y 25095790 PMC4149208

[B23] Green A. Cheong P. W. Fook-Chong S. Tiruchelvarayan R. Guo C. M. Yue W. M. (2015). Cortical reorganization is associated with surgical decompression of cervical spondylotic myelopathy. *Neural Plast.* 2015:389531. 10.1155/2015/389531 26609437 PMC4644848

[B24] Guez M. (2006). Chronic neck pain. An epidemiological, psychological and SPECT study with emphasis on whiplash-associated disorders. *Acta Orthop. Suppl.* 77 preceding 1 3–33. 16544560

[B25] Hahn T. Kircher T. Straube B. Wittchen H. U. Konrad C. Strohle A. (2015). Predicting treatment response to cognitive behavioral therapy in panic disorder with agoraphobia by integrating local neural information. *JAMA Psychiatry* 72 68–74. 10.1001/jamapsychiatry.2014.1741 25409415

[B26] Hart H. Chantiluke K. Cubillo A. I. Smith A. B. Simmons A. Brammer M. J. (2014). Pattern classification of response inhibition in ADHD: toward the development of neurobiological markers for ADHD. *Hum. Brain Mapp.* 35 3083–3094. 10.1002/hbm.22386 24123508 PMC4190683

[B27] Haxby J. V. (2012). Multivariate pattern analysis of fMRI: the early beginnings. *Neuroimage* 62 852–855. 10.1016/j.neuroimage.2012.03.016 22425670 PMC3389290

[B28] Hojat M. Cohen M. J. (2012). “Physicians’ perception of pain as related to empathy, sympathy and the mirror-neuron system,” in *Culture, Brain, and Analgesia: Understanding and Managing Pain in Diverse Populations*, 1st Edn, eds Incayawar M. Todd K. H. (Oxford: Oxford University Press), 63–74.

[B29] Holly L. T. (2009). Management of cervical spondylotic myelopathy with insights from metabolic imaging of the spinal cord and brain. *Curr. Opin. Neurol.* 22 575–581. 10.1097/WCO.0b013e3283325ea7 19741530

[B30] Holly L. T. Dong Y. Albistegui-DuBois R. Marehbian J. Dobkin B. (2007). Cortical reorganization in patients with cervical spondylotic myelopathy. *J. Neurosurg. Spine* 6 544–551. 10.3171/spi.2007.6.6.5 17561743 PMC4160311

[B31] Hotz-Boendermaker S. Marcar V. L. Meier M. L. Boendermaker B. Humphreys B. K. (2016). Reorganization in secondary somatosensory cortex in chronic low back pain patients. *Spine* 41 E667–E673. 10.1097/BRS.0000000000001348 27244113

[B32] Hoy D. G. Protani M. De R. Buchbinder R. (2010). The epidemiology of neck pain. *Best Pract. Res. Clin. Rheumatol.* 24 783–792. 10.1016/j.berh.2011.01.019 21665126

[B33] Hrabalek L. Hlustik P. Hok P. Cechakova E. Wanek T. Otruba P. (2015). [Influence of cervical spondylotic spinal cord compression on cerebral cortical adaptation. radiological study]. *Acta Chir. Orthop. Traumatol. Cech.* 82 404–411. 26787180

[B34] Hrabalek L. Hlustik P. Hok P. Wanek T. Otruba P. Cechakova E. (2014). [Effects of spinal cord decompression in patients with cervical spondylotic myelopathy oncortical brain activations]. *Rozhl Chir.* 93 530–535. 25418940

[B35] Huang T. Zhao Z. Yan C. Lu J. Li X. Tang C. (2016). Altered spontaneous activity in patients with persistent somatoform pain disorder revealed by regional homogeneity. *PLoS One* 11:e0151360. 10.1371/journal.pone.0151360 26977802 PMC4792417

[B36] Iacoboni M. Dapretto M. (2006). The mirror neuron system and the consequences of its dysfunction. *Nat. Rev. Neurosci.* 7 942–951. 10.1038/nrn2024 17115076

[B37] Ke J. Qi R. Liu C. Xu Q. Wang F. Zhang L. (2015). Abnormal regional homogeneity in patients with irritable bowel syndrome: a resting-state functional MRI study. *Neurogastroenterol. Motil.* 27 1796–1803. 10.1111/nmo.12692 26403620

[B38] Kendall M. G. (1990). *Rank Correlation Methods.* Oxford: Oxford University Press.

[B39] Kippenhan J. S. Barker W. W. Pascal S. Nagel J. Duara R. (1992). Evaluation of a neural-network classifier for PET scans of normal and Alzheimer’s disease subjects. *J. Nucl. Med.* 33 1459–1467. 1634935

[B40] Kong J. Spaeth R. B. Wey H. Y. Cheetham A. Cook A. H. Jensen K. (2013). S1 is associated with chronic low back pain: a functional and structural MRI study. *Mol. Pain* 9:43. 10.1186/1744-8069-9-43 23965184 PMC3765748

[B41] Kowalczyk I. Duggal N. Bartha R. (2012). Proton magnetic resonance spectroscopy of the motor cortex in cervical myelopathy. *Brain* 135 461–468. 10.1093/brain/awr328 22180462

[B42] Krall S. C. Rottschy C. Oberwelland E. Bzdok D. Fox P. T. Eickhoff S. B. (2015). The role of the right temporoparietal junction in attention and social interaction as revealed by ALE meta-analysis. *Brain Struct. Funct.* 220 587–604. 10.1007/s00429-014-0803-z 24915964 PMC4791048

[B43] Leak A. M. Cooper J. Dyer S. Williams K. A. Turner-Stokes L. Frank A. O. (1994). The northwick park neck pain questionnaire, devised to measure neck pain and disability. *Br. J. Rheumatol.* 33 469–474. 10.1093/rheumatology/33.5.469 8173853

[B44] Li J. Zhang J. H. Yi T. Tang W. J. Wang S. W. Dong J. C. (2014). Acupuncture treatment of chronic low back pain reverses an abnormal brain default mode network in correlation with clinical pain relief. *Acupunct. Med.* 32 102–108. 10.1136/acupmed-2013-010423 24280949

[B45] Li Z. Liu M. Lan L. Zeng F. Makris N. Liang Y. (2016). Altered periaqueductal gray resting state functional connectivity in migraine and the modulation effect of treatment. *Sci. Rep.* 6:20298. 10.1038/srep20298 26839078 PMC4738255

[B46] Liang M. J. Zhou Q. Yang K. R. Yang X. L. Fang J. Chen W. L. (2013). Identify changes of brain regional homogeneity in bipolar disorder and unipolar depression using resting-state FMRI. *PLoS One* 8:e79999. 10.1371/journal.pone.0079999 24324588 PMC3851159

[B47] Linnman C. Appel L. Soderlund A. Frans O. Engler H. Furmark T. (2009). Chronic whiplash symptoms are related to altered regional cerebral blood flow in the resting state. *Eur. J. Pain* 13 65–70. 10.1016/j.ejpain.2008.03.001 18486506

[B48] Loggia M. L. Kim J. Gollub R. L. Vangel M. G. Kirsch I. Kong J. (2013). Default mode network connectivity encodes clinical pain: an arterial spin labeling study. *Pain* 154 24–33. 10.1016/j.pain.2012.07.029 23111164 PMC3534957

[B49] Lopez-Sola M. Woo C. W. Pujol J. Deus J. Harrison B. J. Monfort J. (2017). Towards a neurophysiological signature for fibromyalgia. *Pain* 158 34–47. 10.1097/j.pain.0000000000000707 27583567 PMC5161739

[B50] Lorberboym M. Gilad R. Gorin V. Sadeh M. Lampl Y. (2002). Late whiplash syndrome: correlation of brain SPECT with neuropsychological tests and P300 event-related potential. *J. Trauma* 52 521–526. 10.1097/00005373-200203000-00017 11901329

[B51] Lorimer Moseley G. (2005). Widespread brain activity during an abdominal task markedly reduced after pain physiology education: fMRI evaluation of a single patient with chronic low back pain. *Aust. J. Physiother.* 51 49–52. 10.1016/S0004-9514(05)70053-2 15748125

[B52] Louw A. Puentedura E. J. Diener I. Peoples R. R. (2015). Preoperative therapeutic neuroscience education for lumbar radiculopathy: a single-case fMRI report. *Physiother. Theory Pract.* 31 496–508. 10.3109/09593985.2015.1038374 26395827

[B53] Mao C. P. Zhang Q. L. Bao F. X. Liao X. Yang X. L. Zhang M. (2014). Decreased activation of cingulo-frontal-parietal cognitive/attention network during an attention-demanding task in patients with chronic low back pain. *Neuroradiology* 56 903–912. 10.1007/s00234-014-1391-6 24986218

[B54] Mars R. B. Sallet J. Schuffelgen U. Jbabdi S. Toni I. Rushworth M. F. (2012). Connectivity-based subdivisions of the human right “temporoparietal junction area”: evidence for different areas participating in different cortical networks. *Cereb. Cortex* 22 1894–1903. 10.1093/cercor/bhr268 21955921

[B55] Orru G. Pettersson-Yeo W. Marquand A. F. Sartori G. Mechelli A. (2012). Using support vector machine to identify imaging biomarkers of neurological and psychiatric disease: a critical review. *Neurosci. Biobehav. Rev.* 36 1140–1152. 10.1016/j.neubiorev.2012.01.004 22305994

[B56] Otte A. Ettlin T. Fierz L. Mueller-Brand J. (1996). Parieto-occipital hypoperfusion in late whiplash syndrome: first quantitative SPET study using technetium-99m bicisate (ECD). *Eur. J. Nucl. Med.* 23 72–74. 10.1007/BF01736993 8586106

[B57] Otte A. Ettlin T. M. Nitzsche E. U. Wachter K. Hoegerle S. Simon G. H. (1997a). PET and SPECT in whiplash syndrome: a new approach to a forgotten brain? *J. Neurol Neurosurg. Psychiatry* 63 368–372. 9328255 10.1136/jnnp.63.3.368 PMC2169690

[B58] Otte A. Mueller-Brand J. Fierz L. (1995). Brain SPECT findings in late whiplash syndrome. *Lancet* 345:1513 10.1016/S0140-6736(95)91075-1 7769927

[B59] Otte A. Mueller-Brand J. Nitzsche E. U. Wachter K. Ettlin T. M. (1997b). Functional brain imaging in 200 patients after whiplash injury. *J. Nucl. Med.* 38:1002. 9189162

[B60] Pereira F. Mitchell T. Botvinick M. (2009). Machine learning classifiers and fMRI: a tutorial overview. *Neuroimage* 45 S199–S209. 10.1016/j.neuroimage.2008.11.007 19070668 PMC2892746

[B61] Pijnenburg M. Brumagne S. Caeyenberghs K. Janssens L. Goossens N. Marinazzo D. (2015). Resting-state functional connectivity of the sensorimotor network in individuals with nonspecific low back pain and the association with the sit-to-stand-to-sit task. *Brain Connect.* 5 303–311. 10.1089/brain.2014.0309 25557846

[B62] Ploner M. Schmitz F. Freund H. J. Schnitzler A. (1999). Parallel activation of primary and secondary somatosensory cortices in human pain processing. *J. Neurophysiol.* 81 3100–3104. 10.1152/jn.1999.81.6.3100 10368426

[B63] Ramachandran V. S. Oberman L. M. (2006). Broken mirrors: a theory of autism. *Sci. Am.* 295 62–69. 10.1038/scientificamerican1106-62 17076085

[B64] Rizzolatti G. Sinigaglia C. (2010). The functional role of the parieto-frontal mirror circuit: interpretations and misinterpretations. *Nat. Rev. Neurosci.* 11 264–274. 10.1038/nrn2805 20216547

[B65] Rodriguez-Raecke R. Niemeier A. Ihle K. Ruether W. May A. (2009). Brain gray matter decrease in chronic pain is the consequence and not the cause of pain. *J. Neurosci.* 29 13746–13750. 10.1523/JNEUROSCI.3687-09.2009 19889986 PMC6666725

[B66] Sarpal D. K. Argyelan M. Robinson D. G. Szeszko P. R. Karlsgodt K. H. John M. (2016). Baseline striatal functional connectivity as a predictor of response to antipsychotic drug treatment. *Am. J. Psychiatry* 173 69–77. 10.1176/appi.ajp.2015.14121571 26315980 PMC4845897

[B67] Sato M. Inubushi M. Shiga T. Hirata K. Okamoto S. Kamibayashi T. (2009). Therapeutic effects of acupuncture in patients with rheumatoid arthritis: a prospective study using (18)F-FDG-PET. *Ann. Nucl. Med.* 23 311–316. 10.1007/s12149-009-0238-4 19337783

[B68] Seminowicz D. A. Wideman T. H. Naso L. Hatami-Khoroushahi Z. Fallatah S. Ware M. A. (2011). Effective treatment of chronic low back pain in humans reverses abnormal brain anatomy and function. *J. Neurosci.* 31 7540–7550. 10.1523/JNEUROSCI.5280-10.2011 21593339 PMC6622603

[B69] Shen H. Wang L. Liu Y. Hu D. (2010). Discriminative analysis of resting-state functional connectivity patterns of schizophrenia using low dimensional embedding of fMRI. *Neuroimage* 49 3110–3121. 10.1016/j.neuroimage.2009.11.011 19931396

[B70] Shi Y. Liu Z. Zhang S. Li Q. Guo S. Yang J. (2015). Brain network response to acupuncture stimuli in experimental acute low back pain: an fMRI study. *Evid. Based Complement. Alternat. Med.* 2015:210120. 10.1155/2015/210120 26161117 PMC4487721

[B71] Tam S. Barry R. L. Bartha R. Duggal N. (2010). Changes in functional magnetic resonance imaging cortical activation after decompression of cervical spondylosis: case report. *Neurosurgery* 67 E863–E864. 10.1227/01.NEU.0000374848.86299.17 20657323

[B72] Tan Y. Zhou F. Wu L. Liu Z. Zeng X. Gong H. (2015). Alteration of regional homogeneity within the sensorimotor network after spinal cord decompression in cervical spondylotic myelopathy: a resting-state fMRI study. *Biomed. Res Int.* 2015:647958. 10.1155/2015/647958 26605335 PMC4641924

[B73] Tang C. C. Poston K. L. Eckert T. Feigin A. Frucht S. Gudesblatt M. (2010). Differential diagnosis of parkinsonismml: a metabolic imaging study using pattern analysis. *Lancet Neurol.* 9 149–158. 10.1016/S1474-4422(10)70002-8 20061183 PMC4617666

[B74] Tsakiris M. Costantini M. Haggard P. (2008). The role of the right temporo-parietal junction in maintaining a coherent sense of one’s body. *Neuropsychologia* 46 3014–3018. 10.1016/j.neuropsychologia.2008.06.004 18601939

[B75] Tu Y. Tan A. Bai Y. Hung Y. S. Zhang Z. (2016). Decoding subjective intensity of nociceptive pain from pre-stimulus and post-stimulus brain activities. *Front. Comput. Neurosci.* 10:32. 10.3389/fncom.2016.00032 27148029 PMC4830829

[B76] Tu Y. H. Fu Z. N. Tan A. Huang G. Hu L. Hung Y. S. (2017). A novel and effective fMRI decoding approach based on sliced inverse regression and its application to pain prediction. *Neurocomputing* 273 373–384. 10.1016/j.neucom.2017.07.045

[B77] Ung H. Brown J. E. Johnson K. A. Younger J. Hush J. Mackey S. (2014). Multivariate classification of structural MRI data detects chronic low back pain. *Cereb. Cortex* 24 1037–1044. 10.1093/cercor/bhs378 23246778 PMC3948494

[B78] Vrana A. Hotz-Boendermaker S. Stampfli P. Hanggi J. Seifritz E. Humphreys B. K. (2015). Differential neural processing during motor imagery of daily activities in chronic low back pain patients. *PLoS One* 10:e0142391. 10.1371/journal.pone.0142391 26569602 PMC4646462

[B79] Wand B. M. Parkitny L. O’Connell N. E. Luomajoki H. McAuley J. H. Thacker M. (2011). Cortical changes in chronic low back pain: current state of the art and implications for clinical practice. *Man. Ther.* 16 15–20. 10.1016/j.math.2010.06.008 20655796

[B80] Wang P. Du H. Chen N. Guo J. Gong Q. Zhang J. (2014). Regional homogeneity abnormalities in patients with tension-type headache: a resting-state fMRI study. *Neurosci. Bull.* 30 949–955. 10.1007/s12264-013-1468-6 25098351 PMC5562557

[B81] Wang Y. Zhang X. Guan Q. Wan L. Yi Y. Liu C. F. (2015). Altered regional homogeneity of spontaneous brain activity in idiopathic trigeminal neuralgia. *Neuropsychiatr. Dis. Treat.* 11 2659–2666. 10.2147/NDT.S94877 26508861 PMC4610767

[B82] Wasan A. D. Loggia M. L. Chen L. Q. Napadow V. Kong J. Gollub R. L. (2011). Neural correlates of chronic low back pain measured by arterial spin labeling. *Anesthesiology* 115 364–374. 10.1097/ALN.0b013e318220e880 21720241 PMC3286828

[B83] Whitfield-Gabrieli S. Ghosh S. S. Nieto-Castanon A. Saygin Z. Doehrmann O. Chai X. J. (2016). Brain connectomics predict response to treatment in social anxiety disorder. *Mol. Psychiatry* 21 680–685. 10.1038/mp.2015.109 26260493

[B84] Woo C. W. Chang L. J. Lindquist M. A. Wager T. D. (2017). Building better biomarkers: brain models in translational neuroimaging. *Nat. Neurosci.* 20 365–377. 10.1038/nn.4478 28230847 PMC5988350

[B85] Yang H. Li L. Peng H. Liu T. Young A. H. Angst J. (2016). Alterations in regional homogeneity of resting-state brain activity in patients with major depressive disorder screening positive on the 32-item hypomania checklist (HCL-32). *J. Affect. Disord.* 203 69–76. 10.1016/j.jad.2016.05.004 27280965

[B86] Yang X. Y. Sun J. Luo J. Zhong Z. X. Li P. Yao S. M. (2015). Regional homogeneity of spontaneous brain activity in adult patients with obsessive-compulsive disorder before and after cognitive behavioural therapy. *J. Affect. Disord.* 188 243–251. 10.1016/j.jad.2015.07.048 26378734

[B87] Ye Z. Rae C. L. Nombela C. Ham T. Rittman T. Jones P. S. (2016). Predicting beneficial effects of atomoxetine and citalopram on response inhibition in Parkinson’s disease with clinical and neuroimaging measures. *Hum. Brain Mapp.* 37 1026–1037. 10.1002/hbm.23087 26757216 PMC4819701

[B88] Younger J. W. Chu L. F. D’Arcy N. T. Trott K. E. Jastrzab L. E. Mackey S. C. (2011). Prescription opioid analgesics rapidly change the human brain. *Pain* 152 1803–1810. 10.1016/j.pain.2011.03.028 21531077 PMC3138838

[B89] Yu R. Gollub R. L. Spaeth R. Napadow V. Wasan A. Kong J. (2014a). Disrupted functional connectivity of the periaqueductal gray in chronic low back pain. *Neuroimage Clin.* 6 100–108. 10.1016/j.nicl.2014.08.019 25379421 PMC4215524

[B90] Yu R. Gollub R. L. Vangel M. Kaptchuk T. Smoller J. W. Kong J. (2014b). Placebo analgesia and reward processing: integrating genetics, personality, and intrinsic brain activity. *Hum. Brain Mapp.* 35 4583–4593. 10.1002/hbm.22496 24578196 PMC4107077

[B91] Zang Y. Jiang T. Lu Y. He Y. Tian L. (2004). Regional homogeneity approach to fMRI data analysis. *Neuroimage* 22 394–400. 10.1016/j.neuroimage.2003.12.030 15110032

[B92] Zeng L. L. Shen H. Liu L. Wang L. Li B. Fang P. (2012). Identifying major depression using whole-brain functional connectivity: a multivariate pattern analysis. *Brain* 135 1498–1507. 10.1093/brain/aws059 22418737

[B93] Zhang J. Su J. Wang M. Zhao Y. Yao Q. Zhang Q. (2016). Increased default mode network connectivity and increased regional homogeneity in migraineurs without aura. *J. Headache Pain* 17:98. 27771875 10.1186/s10194-016-0692-z PMC5075323

[B94] Zhou F. Gong H. Liu X. Wu L. Luk K. D. Hu Y. (2014). Increased low-frequency oscillation amplitude of sensorimotor cortex associated with the severity of structural impairment in cervical myelopathy. *PLoS One* 9:e104442. 10.1371/journal.pone.0104442 25111566 PMC4128667

[B95] Zhou F. Wu L. Liu X. Gong H. Luk K. D. Hu Y. (2015). Characterizing thalamocortical disturbances in cervical spondylotic myelopathy: revealed by functional connectivity under two slow frequency bands. *PLoS One* 10:e0125913. 10.1371/journal.pone.0125913 26053316 PMC4460123

[B96] Zhou F. Q. Tan Y. M. Wu L. Zhuang Y. He L. C. Gong H. H. (2015). Intrinsic functional plasticity of the sensory-motor network in patients with cervical spondylotic myelopathy. *Sci. Rep.* 5:9975. 10.1038/srep09975 25897648 PMC4404678

[B97] Zhou Y. Jin J. (2008). Effect of acupuncture given at the HT 7, ST 36, ST 40 and KI 3 acupoints on various parts of the brains of Alzheimer’ s disease patients. *Acupunct. Electrother. Res.* 33 9–17. 10.3727/036012908803861186 18672741

